# Awareness, practices and perspectives on ensuring access to ideally packaged iodized salt in Nigeria

**DOI:** 10.1016/j.dialog.2023.100148

**Published:** 2023-07-20

**Authors:** Oluwaseun Ariyo, Opeyemi Akintimehin, Anuoluwapo Funmilayo Taiwo, Thelma Nwandu, Bukola Olanrewaju Olaniyi

**Affiliations:** a Department of Human Nutrition and Dietetics, University of Ibadan, Nigeria; b World Bank Accelerating Nutrition Results in Nigeria Project, Lokoja, Kogi State, Nigeria

**Keywords:** Iodized salt, Stakeholders perspective, Proper food packaging, Open-air salt Universal Salt Iodization taskforce

## Abstract

Salt iodization is a positive exemplar of a sustainable public-private partnership in promoting better nutrition outcomes in many countries. However, the gains in the past decades are gradually being eroded, following laxity in policy implementation, monitoring and regulatory roles resulting in increasing access to non-labelled salt in the Nigerian market. This study was designed to evaluate the awareness, practices and perspectives on salt iodization and regulations among salt marketers and consumers in Ibadan, Oyo state.

This mixed-method study was carried out in seven major markets across Ibadan metropolis. A three-stage sampling technique was used to select 77 salt users/clients, 103 salt vendors, 12 salt wholesalers and four regulators/producers. Interviewer-administered questionnaire was used to collect information on types/brands of salt, handling, retail practices, awareness, and salt purchase preference. Structured in-depth interview was used to elicit information on existing regulations, compliance level, and monitoring activities. Descriptive statistics were used to analyse quantitative data. Interviews were tape-recorded, transcribed verbatim and analyzed thematically.

Males constituted 66.7%, 1.0% and 14.3% of respondents among wholesalers, retail vendors, and clients, respectively, with 100.0%, 58.3% and 84.4% having at least primary education. All wholesalers and 30.1% of retail vendors used shaded structure. About 67% of the wholesalers and 58.3% of the retailers sold branded salt. Clients’ basis for the use of non-branded salt included cheapness and greater quantity (54.5%), higher intensiveness/saltiness and greater quantity (22.1%), and cheaper cost (18.2%). Only 3% of the consumers were aware of mandatory salt iodization, 3.9% were aware of guidelines on salt marketing and only 18.2% handled salt safely. Safe handling practices were found among all wholesalers and 44.7% of the retailers. Qualitative findings revealed the existence of regulation on the production, packing and marketing of salts in Nigeria, however, enforcement and monitoring at the market level is weak.

The demand and use of industrial salt in food preparation remain widespread among consumers in Ibadan, Nigeria following limited awareness of salt iodization programme and its benefits. Regulations on salt marketing should be enforced at all levels and nutrition education on salt iodization should be intensified.

## 1 Introduction

Universal salt iodization is one of the positive exemplars of food fortification globally. The strategy has been commended for reducing the global cases of clinical Iodine Deficiency Disorders (IDDs) by 75.9% and improving cognitive development and future earnings with economic benefit of nearly $33 billion [13,22]. In Nigeria, universal salt iodization was considered largely successful following adequate fortification of over 90% of domestic salt within ten years of implementation. Studies have found continuous compliance with the recommended level of 30ppm in some Nigerian retail common branded salts, however, iodine is either completely absent or lower than recommended in non-branded salts [1,11,15]. Interestingly, the market share of non-branded/non-iodised salt has been on the rise in Nigeria from less than five percent in 1994 to about 25 percent presently. Presently, West Africa including Nigeria has the lowest coverage of iodised salt and about 25% of the population consumes salt without any iodine [21]. Pockets of studies within the country have put the proportion of household consuming salt without iodine at about 30 percent [1,20].

In Nigeria, policy, regulation, and enforcement process have been integrated in the salt iodization system with strong public private partnerships. Despite these measures, the gains of the past years have gradually been eroded following weak monitoring. Measures to ensure effectiveness and sustainability of universal salt iodization programme in Nigeria included the establishment of the iodine deficiency – Universal Salt Iodization taskforce and multi-sectoral reporting channels; a ban on domestic salt packaging in 25kg sacks and introduction of mandatorily packaged salt in small retail sizes (1kg and less) to enhance retention of quality iodine and content and ban on marketing or hawking of salt in non-shaded stalls [4]. These measures enable consumers to store salt in proper containers at home and distinguish between salts for industrial and domestic use. Yet, increasing cases of marketing of salts in household measures and in open spaces have been observed in recent times. Akinola et al. [3] reported increasing access of consumers to non-branded salt in Nigeria’s open markets. These practices have implications on the iodide content of domestic salt, and ultimately on the health and well-being of the consumers. Continued deprivation of the segment of the population access to adequate iodine intake through universal salt iodization has consequences on the burden of stunting, intelligence quotient and cognition, and workforce productivity. Evidence has shown that iodised salt helps children achieve 8.18% increased intelligence quotient [13], 1.85cm increase in height [18], 4.81% and 2.67% increase in basic numeracy skills for girls and boys, respectively [18] and 1.2 percentage points increase in labor force participation among women, and 11% increase in total income [2].

Reversing the downward trends of universal salt iodization in Nigeria requires involvement of all stakeholders across the salt supply chain including the salt producing companies, marketers, government regulatory bodies, and consumers. Presently, there is a dearth of information on what motivates the increasing marketing and patronage of non-branded salts in Nigeria. Understanding this reasoning is important in promoting appropriate strategies to increase uptake and use of iodized salt and strengthen universal salt iodization programme in Ibadan specifically, and Nigeria at large.

To this end, this study was designed to evaluate the awareness, practices and perspectives on salt iodization and regulations among salt marketers and consumers in Ibadan, Oyo state.

## 2 Methodology

### 2.1 Study design

This study employed an exploratory sequential mixed-method design.

### 2.2 Study area

The study was carried out in Ibadan, Oyo State. Ibadan lies within latitude 7° 19’ 08” and 7° 29’ 25” of the equator and longitude 3° 47’ 50” and 4° 0’ 22” and is located in the South-West, Nigeria. It is a largely cosmopolitan town and the capital of Oyo State and consist of nine out of the 33 Local Government Areas (LGAs) in the state. Oyo State has an equatorial climate with dry and wet seasons and relatively high humidity. The projected population of Ibadan is about 3,875,000.

### 2.3 Study location

The study covered seven major markets across six Local Government Areas (LGAs) in Ibadan. These markets are Bodija market in Ibadan North, Oje market in Ibadan North-East, Oja-Oba and Bode markets in Ibadan South-East, Alesinloye market in Ibadan South-West, Agbeni market in Ibadan North-West and Gbagi market in Egbeda LGAs.

### 2.4 Study population

Five set of study population consisting of stakeholders in the domestic salt supply chain in Ibadan were used for the study. The population included officials of the government regulatory/enforcement agencies such as National Agency for Food and Drug Administration and Control (NAFDAC), Standards Organization of Nigeria (SON), and Federal Competition and Consumer Protection Commission (FCCPC); industries producing salt in 25kg sacs and more (NASCON Allied Limited Plc and Royal Salt), major wholesalers/distributors of salt in Ibadan, salt retailers and vendors in sampled markets and their clients or end users.

### 2.5 Sampling techniques

A three-stage sampling procedure was adopted for the study. In the first stage, the six major food markets in the city of Ibadan were purposively selected. Rapid mapping of salt retailers/vendors (branded salt, unpackaged salt, or both) across the six markets was conducted and quota sampling was adopted to select 103 salt retailers including 20, 20, 10, 15, 10, 8, and 20 from Bodija, Oje, Gbagi, Oja-Oba, Bode, Alesinloye and Agbeni, respectively. Using snowball sampling and referral from the salt retailers, 12 salt wholesalers were sampled across four markets including Agbeni (5), Bodija (3), Oje (2) and Gbagi markets (2). Also, purposive sampling of 77 consumers/end users that patronized retailers across three markets, Oje (27), Bodija (25), and Agbeni (25) was adopted. For the regulatory/enforcement agencies, purposive sampling of officials with at least five years working experience and member of Iodine Deficiency-Universal Salt Iodization Taskforce was adopted.

### 2.6 Data collection procedure

Data were collected between August and November 2021; using purposive sampling of major markets from six selected local governments in Ibadan, Oyo state. Listing of salt retailers, customer and vendor was carried out, to determine the number of stakeholders at the market level. Retailers who sold branded packaged and unpackaged salts were listed out. From the listing, quota sampling of retailers who sold packaged branded packaged salt and unpackaged salt together and unpackaged salt only were selected. Target Customers were selected based on the purchase of the unpackaged salt from retailers and was carried out in three markets (Bodija, Agbeni and Oje market). Data collection from these target customers was done from retailers’ standpoint who sold branded packaged salt and unpackaged salt together. Vendors (Wholesaler) were selected based on the conversation from the retailers and observations.

### 2.7 Quantitative data

Data were collected using a semi-structured, interviewer-administered questionnaire for the salt retailers/vendors and consumers/end-users. Observational checklist was used to record the type and brand of the salt where applicable, method of storage, texture and crystal sizes of the salt samples categorised as fine, coarse, and granular. Questionnaire administered to the retailers and wholesalers was designed to collect information on the source of supply/purchase, average quantity sold per day, average hours spent in the market per day and awareness of regulation on salt marketing. Consumers’ questionnaire was used to collect information on the awareness of branded, packaged salt, consumers satisfaction, salt purchase preference and handling of salt. Safe handling of salt was defined as storage of salt in closed container and away from heat. All the research instruments were checked for completeness and internal consistency. Quantitative data were analysed and presented using descriptive statistics, largely frequencies and percentages.

### 2.8 Qualitative data

Key In-depth interview was conducted using a semi-structured guide for ten officials of salt-producing industries and regulatory/enforcement agencies to elicit information on existing regulations, compliance to regulations on salt iodization and marketing, and monitoring activities in Nigeria and strategies to promote consumers awareness. Respondents were drawn from the National Agency for Food and Drug Administration and Control, Standard of Organisation Nigeria, the Federal Competition and Consumer Protection Commission, and Salt industries that produce salt in 25kg sacs and more (Royal Salt and Dangote). Standard demographic data such as age of the respondents, marital status, education level and years of working experience were also collected. All interviews were conducted in a serene and comfortable environment devoid of distraction to aid stakeholders in sharing their perspectives and experiences. Each interview session lasted about 45-60 minutes and the sessions were recorded with participants’ consent. Recorded interviews were transcribed verbatim and triangulated for content confirmation. The standard six-step procedure including familiarization, coding, generating themes, reviewing themes, defining, and naming themes and writing up was adopted for thematic analysis [6]. The data were manually coded using an inductive, bottom-up, data-driven approach that avoids fitting the data to existing frameworks or preconceived categories. Themes were validated by a second researcher. Five categorical codes were generated including “regulation”, “inspection”, “monitoring”, “awareness” and “education”. Themes were then generated from these codes; these themes represented information that appeared to provide authentication of the experiences and perspectives of these stakeholders. Themes were then reanalyzed, verified, and validity was established using peer debriefing and confirmability [7,14].

### 2.9 Ethical consideration

Oral informed consent was obtained from all the respondents. Anonymity and confidentiality of the information were ensured. The study was approved by the University of Ibadan/ University College Hospital Research and Ethics Committee (UI/EC/21/0673). The study complied with all appropriate rules and regulations as established by the ethics committee.

## 3 Results

### 3.1 Basic characteristics of marketers and consumers

The basic characteristics of the stakeholders sampled for the study is presented in Table 1. Male respondents accounted for 66.7%, 1.0% and 14.3% of the wholesalers, retail vendors, and clients / end users, respectively. Sixty percent of the wholesalers were age 41-60 years and 25.0% were 21-40 years old. Most wholesalers (91.6%) had at least secondary education, retail vendors largely had no formal education (41.7%) or only primary education (27.2%) while only 15.6% of the end users had no formal education. About 30 percent of the end users were traders, 19.5% were food vendors, 15.6% were traders and other occupations accounted for 35.1%. All the wholesalers (100.0%) transacted business in a shaded structure while majority (69.9%) of retail vendors operated without shaded structure. In addition, 50% of the wholesalers’ traded in salt for both domestic and industrial use, 33.3% traded in industrial salt and 16.7% in salt for domestic use.

**Table 1:** Sociodemographic characteristic of sampled stakeholders.

	Wholesalers		Retail Vendors		Clients	
	N=12		N=103	%	N =77	%
Gender						
Male	8	66.7	1	1.0	11	14.3
Female	4	33.3	102	99.0	66	85.7
Age						
>20	0	0.0				
21 – 40	3	25.0				
41 – 60	7	58.3				
>60	2	16.7				
Education						
No Formal Education	0	0.0	43	41.7	12	15.6
Primary Education	1	8.3	28	27.2	13	16.9
Secondary Education Uncompleted	0	0.0	19	18.4	19	24.7
Secondary Education Completed	4	33.3	13	12.6	26	33.8
Tertiary Education	7	58.3			7	9.1
Marital Status						
Married	12	100.0	12	100.0	61	79.2
Single					14	18.2
Widowed					2	2.6
Major Occupation	-	-	-	-		
Food Vendor	-	-	-	-	15	19.5
Artisan	-	-	-	-	23	29.9
Trader	12	100.0	12	100.0	12	15.6
Others	-	-	-	-	27	35.1
Type of Retail Outlet						
No Shop/ No shade	-	-	72	69.9	-	-
Shaded Stall/Shop	12	100.0	31	30.0	-	-
Type of Salt Being Sold						
Domestic	2	16.7				
Industrial	4	33.3				
Domestic/Industrial	6	50.0				

### 3.2 Consumers’ awareness and practices on salt purchase and use

In Table 2, consumers’ awareness, and practices with respect to salt purchase and use are presented. The major basis for the use of non-branded salt included relative cheapness and greater quantity (54.5%), higher intensiveness/saltiness and greater quantity (22.1%), and relative cheaper cost (18.2%). About half (49.4%) purchased non-branded salt weekly, 11.7% monthly and 39% occasionally. The most common quantity purchased at a time was 3-5 cups, approximately 660-1100g (45.5%), 1-2 cups, about 220-440g (36.4%) and 1-2 Kongos, 2,200-4,400g (18.2%). Major uses to which the salt was applied included household cooking (76.6%) and food catering services (16.9%). Only 3 percent of the consumers were aware of mandatory salt iodization, 3.9% were aware of guidelines on salt marketing and only 18.2% handled salt safely.

**Table 2:** Consumers/end users Awareness, practices, and Use of salt.

	N =77	%
Basis for use of non-branded salt		
Relatively cheaper	14	18.2
Greater quantity	4	5.2
Relative cheaper & greater quantity	42	54.5
Intensiveness & greater quantity	17	22.1
Frequency of Purchase		
Monthly	9	11.7
Weekly	38	49.4
Occasionally	30	39.0
Quantity usually purchased per time		
1-2 *Kongo*⁎ (2,200-4,400g)	14	18.2
3-5 milk tin (660-1100g)	35	45.5
1-2 milk tin (220-440g)	28	36.4
Use of the Salt Purchased		
Household Cooking	59	76.6
Food Catering Services	13	16.9
Others	5	6.5
Awareness of mandatory salt iodization		
Yes	2	2.6
No	75	97.4
Awareness of policy/guideline on salt marketing		
Yes	3	3.9
No	74	96.1
Source of awareness of salt iodization		
None	75	97.4
Advertisement	1	1.3
Internet	**1**	**1.3**
Salt handling practices		
Stored in closed containers	14	18.2
Stored in open containers	4	5.2
Retained in the purchase package	59	76.6

⁎ Kongo is the prevalent unit of measurement in Southwest Nigeria.

### 3.3 Marketers’ salt procurement and handling practices

The practices of the salt wholesalers and retailers in Ibadan are presented in Table 3. About 67 percent of the wholesale marketers and 58.3% of the retail marketers sold branded salt. Among retail marketers, the preference was largely based on consumers higher demand (85.5%). Salt supply for wholesale marketers was largely from 1-2 companies (66.7%) and largely a single company (89.3%) for the retail marketers. Three major company products were identified among the wholesale marketers including NASCON-allied (Dangote), Royal salt and Sweet Nutrition with 50.0%, 33.3%, and 16.7%, respectively. Handling among the wholesale retailers was entirely (100.0%) considered safe while only 44.7% of the retail marketers had safe salt handling practices.

**Table 3:** Marketers practices in salt supply.

	Wholesale Marketers		Retail Marketers	
	N=12		N=103	%
Market branded salt				
Yes	8	66.7	60	58.3
No	4	33.3	43	41.7
Basis for sales preference of non-braded over branded salt				
Higher demand and sales for non-branded salt			88	85.5
Easier supply access and sales			9	8.7
Profit-driven			6	5.8
No. of companies patronized for Salt supply				
1	5	41.7	92	89.3
2	3	25.0	11	10.7
3 or more	4	33.4		
Major sources of salt supply				
NASCON-allied Industry (Dangote industrial salt)	6	50.0		
Royal Salt	4	33.3		
Sweet Nutrition	2	16.7		
Salt handling practices after daily sales	-	-	-	-
Covered the salt bowl with nylon/cloth	-	-	53	51.5
Return/retain salt to sack/sales package	12	100.0	46	44.7
Retained in salt bowl without covering	-	-	4	3.9
Targeted groups for sales				
Industries	3	25.0	-	-
Retailers	9	75.0	-	-
Customers	0	0.0	103	100.0
Sales per #day/week bags				
1-5	3	25.0	⁎103	100.0
6-10	5	41.7		
>10	4	33.4		
Awareness of policy/guideline on salt marketing				
Yes	5	41.7	72	69.9
No	7	58.3	31	30.0
Knew iodized salt is better for health than non-iodized salt				
Yes	5	41.7		
No	7	58.3		

# Sales were per day for wholesalers and per week for retailers.

⁎ Sales per week for retailers were largely 1-2 bags (89.3%).

### 3.4 Perspectives of iodized salt production and regulatory/enforcement stakeholders

A total of ten officials of the regulatory and enforcement agents and salt producing companies was interviewed. The respondents were all male, married, university graduates, belonged to management cadre, and had a mean work experience of 6.0 years. Qualitative findings revealed the existence of regulation on the production, packing and marketing of salts and this was confirmed by all respondents. Some of the participants identified that regulatory agencies focus is largely concentrated on the industry level activities with emphasis on compliance on fortification dosage. “*Yes, there is an existing regulation guiding the sale of salt from the industrial level. We are more involved in salt produced from the industries and the salt sold should be iodized. Fortification of Salt with iodine for both domestic use and industrial use should be done at 50ppm which is stated in the NIS 168: 2004 Standard for Food Grade Salt in Nigeria*” - Official I, SON, Ibadan Office.

There are regulations at the market level for salts, which was established by one of the respondents. “*At market level, there are guidelines in the practice of sale of salt and how retailers have to do to maintain the level of iodine at market level”* – Official I, NAFDAC, Ibadan Office.

This regulation was also confirmed by a respondent from one of the organizations; “*Yes, there are guidelines in the sale of salt from the organisation level.*”- Staff, NASCON Allied Industries.

In addition, the guidelines specify the fortification of salt with iodine for both domestic and industrial use at 50ppm. The guideline detailed the level of fortification expected at the point of production, market, and households’ levels, however, handling and storage conditions could affect the actual level of iodine in salts at the market and household levels. The officials of the salt producing companies confirmed the existence of the guidelines and compliance of the producers with this guideline.

Periodic inspection is conducted by the regulatory agencies to ensure compliance largely at the industry level. “*To ensure salt produced is up to standard at industrial level, SON officials carry out inspections at salt producing industries to ensure the salt produced are up to standard*” – Official II, SON, Ibadan Office.

Sanctions including confiscation of the products are identified as some of the punishment for non-compliance with the regulations. The salt producing companies also confirmed that the regulatory agencies conduct periodic monitoring to ensure compliance with regulations. “The *level of iodine in salt is checked by using a test kit, a blue-black color would appear indicating the presence of iodine. If there is no appearance of color, the product would be confiscated” -* Official II, NAFDAC, Ibadan Office and the salt producing companies.

In addition to the monitoring at the factory or industry level, random sampling of the salt products in the market are also conducted to ascertain the iodine content of the salt at the market level. The National Agency for Food and Drugs Administration and Control usually conducts the market level assessment using test kits. “*Well before the products leave the industry, it is well inspected and analysed to ensure the salt fortification meets its standard. We also carry out inspection at market level, to check its ppm at market level to ensure its not lower than 15ppm. We educated the retailers in the market to ‘please store the product away from the sunlight’. But after awareness it remains a common practice among the retailers.*”- Staff, NASCON Allied Industries.

Findings also show that the salt producing companies engaged in nutrition education of the masses to promote uptake of *purified iodized salt in small sachet from 500g.* The companies erected billboards in strategic locations to educate the masses and encourage the use of fortified salt*. “There* are *billboards advertisement, newspaper campaigns and public notices, regular radio and television jingles, posters, and print materials in strategic areas of the country to create awareness and promote consumption of refined and purified iodized salt in small sachet from 500g below* - Staff, NASCON Allied Industries.

In addition, the producers also engage the vendors on the proper handling and storage of the products, nevertheless compliance to the regulation is low. “*We educated the retailers in the market to ‘please store the product away from the sunlight’. But after awareness it remains a common practice among the retailers.*”- Staff, NASCON Allied Industries.

Nutrition education and awareness had some disruption occasioned by the COVID-19 pandemic, and recovery from this disruption is rather slow. Consequently, nutrition education and awareness have not been regular. - “*There are programs organized to create awareness on the type of product to purchase at both household and market level. But currently those programs have not been scheduled lately due to COVID restriction on crowd gathering and home visitation*”- Official II, NAFDAC, Ibadan Office.

## 4 Discussion

This study evaluated awareness, practices and perspectives on salt iodization and regulations among salt consumers and marketers in Ibadan, Oyo state. It is aimed at generating perspectives to improve consumers access and safe handling of packaged, branded iodized salt. Findings from this study reveals that compliance to recommended salt storage and handling practices at retail and household levels is poor and industrial salt is marketed for domestic use. Also, consumers preference for non-branded salts is high following relative cost advantage and intensiveness over branded salt products, and this informed retail marketers’ stock. Awareness of the benefits of salt iodization is low and poor handling practices is predominant among the retail marketers and the consumers.

Poor storage and handling of salt result in rapid loss of iodine especially when exposed to heat or sunlight [8]. In Nigeria, the poor regulation of food marketing activities favours marketers’ non-compliance with recommended storage practices for many foods including salt. In many local markets, open vending of salt and exposure to direct sunlight, and fluctuating temperature have implications on its iodine content. Emelike et al. [10] reported higher loss of iodine in packaged salt from open markets compared with supermarkets, following differences in storage condition. Greater loss occurs and safety concerns arise with marketing of salt in household measures and non-shaded stalls which are becoming increasingly common in markets around Ibadan, Nigeria. Beside the retailers handling, poor salt handling practices at household level are common in Nigeria including keeping salt close to fire, storage in open container and exposure to sunlight [20].

The increasing access to industrial salt for domestic use constitutes a concern in the country and confirms that access to appropriately iodised salt is declining in Nigeria. In addition, the development re-introduces the unsafe practices of salt marketing in household measures; creates room for marketing of industrial salt for domestic uses and allows for unhygienic practices in salt marketing. Moreover, the practice violates the national recommendation that iodised salt be packaged in airtight, high-density polyethylene or polypropylene or LDPE-lined jute bags and not be exposed to rain, excessive humidity, or direct sunlight at any stage of storage, transportation, or sale [16]. High level of heavy metals and other impurities have been reported in industrial and unrefined salts in Nigeria and its consumption has serious health consequences if unchecked [5,19].

In this study, the consumers preference for non-branded salts and consequently the retail marketers’ stock can be attributed to poor awareness of the benefits of salt iodization. Studies in Nigeria had reported poor knowledge of salt iodization and benefits of iodized salt over its alternatives [9,20]. Though qualitative findings revealed that the salt industries engage in nutrition education and other behaviour change communication interventions, it is obvious that this is not yet impactful, or the coverage is low. The recent disruption of the nutrition education by the COVID pandemic notwithstanding, benefits of salt iodization should be well known with about 30 years of implementation. There may be a need for a systematic approach including the involvement of the marketing stakeholders in the cause of educating the public on the benefits of iodised salts and compliance with recommended handling practices. This is also important following the findings of low awareness of the salt iodization and marketing regulations among both marketers and consumers. Ignorance of the health benefits may contribute to poor safe handling practices and ignorance of the policy and guidelines also encourage non-compliance and reduce social and health impacts of salt iodization programme. Moreover, these marketers need to be provided orientation to see themselves as stakeholders in Nigeria’s achievement and sustenance of universal salt iodization goal. There is a need for public sensitization on government policy and its benefits, need to promote safe handling of salt to preserve the iodine content, improve consumers education on proper handling and recommended keeping condition of salt and other fortified foods.

Likewise, existing regulating, monitoring and punitive activities are concentrated at industry level, extending these activities to the marketing stakeholders is required for improving the coverage and sustaining the gains of universal salt iodization in Nigeria. As such, marketing of industrial salt to the retailers and non-compliance with recommendation should attract heavy sanctions and consumers should be educated to create demand for branded salt in recommended retail packages.

A similar finding has been reported earlier in Nigeria with a call to promote enlightenment campaigns and follow-ups, and constant surveillance at all levels [1]. A double-prong approach of sanitizing the supply chain and demand generation for nutritious products is expected to push the country forward in the use of iodised salt. Evidence from the uptake of biofortified food products have shown that Nigerians are slow in the uptake and use of nutritious food products [12]. However, with improved awareness and understanding of the benefits associated with a product, uptake improves. Oni and Okulaja [17] reported the willingness of consumers to pay for iodine in salt in a Nigeria population. Another key measure is the price regulation to ensure consumers are not discouraged from patronizing branded salt because of large difference in price. One of the key principles in food fortification is ensuring little or no change in price, and adherence of salt iodization implementation to this recommendation is vital in the interest of the public.

## 5 Conclusion

Awareness of salt iodization programme and its benefits remain low among salt marketers and consumers in Ibadan, Nigeria. Consequently, compliance to recommended salt storage and handling practices at retail and household levels is poor, and preference and use of industrial salt in food preparation remains common among consumers. Reversing the gains of universal salt iodization is inevitable if proper action is not initiated. Following this, a nutrition education on the importance and benefits of salt iodization and enforcement of the regulations on salt marketing at all levels are recommended.

## Declaration of Competing Interest

The authors declare that they have no known competing financial interests or personal relationships that could have appeared to influence the work reported in this paper.
